# Supplementary material for: Viral load Reduction in SHIV-Positive Nonhuman Primates via Long-Acting Subcutaneous Tenofovir Alafenamide Fumarate Release from a Nanofluidic Implant
Source: Pharmaceutics. 2020 Oct 17;12(10):981. doi: 10.3390/pharmaceutics12100981 (PMC7590004; doi:10.3390/pharmaceutics12100981)
Supplement: Supplementary file 1 [file pharmaceutics-12-00981-s001.pdf]

pharmaceutics

MDPI

# Supplementary Materials: Viral load Reduction in SHIV-Positive Nonhuman Primates via Long-Acting Subcutaneous Tenofovir Alafenamide Fumarate Release from a Nanofluidic Implant

**Fernanda P. Pons-Faudoa, Nicola Di Trani, Antons Sizovs, Kathryn A. Shelton, Zoha Momin, Lane R. Bushman, Jiaqiong Xu, Dorothy E. Lewis, Sandra Demaria, Trevor Hawkins, James F. Rooney, Mark A. Marzinke, Jason T. Kimata, Peter L. Anderson, Pramod N. Nehete, Roberto C. Arduino, K. Jagannadha Sastry and Alessandro Grattoni**

**Table 1.** Histological characteristics scoring system to evaluate inflammation near the implants.

| **Histological characteristics** | **Score** 0 | 1 | 2 | 3 | 4 | **Reactive Inflammation Multiplier** ($m_{j,r}$) |
|---|---|---|---|---|---|---|
| **Cell characteristic** | | | | | | |
| Polymorphonuclear cells | 0/HPF | Rare, 1-5/HPF | 5-10/HPF | Heavy infiltrate | Packed | 2 |
| Lymphocytes | 0/HPF | Rare, 1-5/HPF | 5-10/HPF | Heavy infiltrate | Packed | 2 |
| Plasma cells | 0/HPF | Rare, 1-5/HPF | 5-10/HPF | Heavy infiltrate | Packed | 2 |
| Macrophages | 0/HPF | Rare, 1-5/HPF | 5-10/HPF | Heavy infiltrate | Packed | 2 |
| Giant cells | 0/HPF | Rare, 1-5/HPF | 5-10/HPF | Heavy infiltrate | Packed | 2 |
| **Tissue characteristic** | | | | | | |
| Necrosis | 0 | Minimal | Mild | Moderate | Severe | 2 |
| Capsule thickness | 0 | Narrow band (<5 cells) | Moderate (5-10 cells) | Thick band (10-20 cells) | Extensive thick band | 1 |
| Tissue infiltrate | 0 | Minimal focal invasion of local tissue | Mild to multifocal inflammation in adjacent tissues | Moderate inflammation in adjacent tissues | Marked inflammation in adjacent tissues | 1 |

Note: Reproduces from [1], ASM Journals, 2020.

**Table 2.** nTAF$_t$ histopathological scoring.

| Animal 15-162 (Treatment 1) | Score | | |
|---|---|---|---|
| | **Pathologist 1** | **Pathologist 2** | **Average** |
| Polymorphonuclear cells | 3 | 2 | **2.5** |
| Lymphocytes | 3 | 1 | **2.0** |
| Plasma cells | 1 | 1 | **1.0** |
| Macrophages | 3 | 3 | **3.0** |
| Giant cells | 1 | 2 | **1.5** |
| Necrosis | 0 | 1 | **0.5** |
| Capsule thickness | 3 | 3 | **3.0** |
| Tissue infiltrate | 1 | 2 | **1.5** |
| **Overall total** | **15** | **15** | **15.0** |
| **Animal 17-048 (Treatment 2)** | **Score** | | |
| | **Pathologist 1** | **Pathologist 2** | **Average** |
| Polymorphonuclear cells | 3 | 0 | **1.5** |
| Lymphocytes | 3 | 1 | **2.0** |
| Plasma cells | 0 | 1 | **0.5** |
| Macrophages | 3 | 3 | **3.0** |
| Giant cells | 0 | 1 | **0.5** |
| Necrosis | 1 | 0 | **0.5** |
| Capsule thickness | 3 | 3 | **3.0** |
| Tissue infiltrate | 2 | 3 | **2.5** |
| **Overall total** | **15** | **12** | **13.5** |
| **Animal 17-071 (Treatment 3)** | **Score** | | |
| | **Pathologist 1** | **Pathologist 2** | **Average** |
| Polymorphonuclear cells | 1 | 1 | **1.0** |
| Lymphocytes | 3 | 2 | **2.5** |
| Plasma cells | 1 | 2 | **1.5** |
| Macrophages | 2 | 3 | **2.5** |
| Giant cells | 0 | 2 | **1.0** |
| Necrosis | 2 | 2 | **2.0** |
| Capsule thickness | 3 | 3 | **3.0** |
| Tissue infiltrate | 1 | 3 | **2.0** |
| **Overall total** | **13** | **18** | **15.5** |
| **Animal 16-131 (Treatment 4)** | **Score** | | |
| | **Pathologist 1** | **Pathologist 2** (Note increase in eosinophils) | **Average** |
| Polymorphonuclear cells | 3 | 2.5 | **2.8** |
| Lymphocytes | 3 | 3 | **3.0** |
| Plasma cells | 1 | 1 | **1.0** |
| Macrophages | 3 | 3 | **3.0** |
| Giant cells | 0 | 1 | **0.5** |
| Necrosis | 3 | 2 | **2.5** |

| | | | |
|---|---|---|---|
| Capsule thickness | 3 | 3 | **3.0** |
| Tissue infiltrate | 1 | 3 | **2.0** |
| **Overall total** | **17** | **18.5** | **17.8** |
| **Animal 15-134 (Treatment 5)** | **Score** | | |
| | **Pathologist 1** | **Pathologist 2** (Note increase in eosinophils) | **Average** |
| Polymorphonuclear cells | 3 | 2 | **2.5** |
| Lymphocytes | 3 | 3 | **3.0** |
| Plasma cells | 2 | 2 | **2.0** |
| Macrophages | 3 | 3 | **3.0** |
| Giant cells | 0 | 1 | **0.5** |
| Necrosis | 4 | 1 | **2.5** |
| Capsule thickness | 4 | 2 | **3.0** |
| Tissue infiltrate | 2 | 2 | **2.0** |
| **Overall total** | **21** | **16** | **18.5** |
| **Animal 17-004 (Treatment 6)** | **Score** | | |
| | **Pathologist 1** | **Pathologist 2** | **Average** |
| Polymorphonuclear cells | NA | 0 | NA |
| Lymphocytes | | 1 | |
| Plasma cells | | 1 | |
| Macrophages | | 1 | |
| Giant cells | | 0 | |
| Necrosis | | 0 | |
| Capsule thickness | NA | 1 | |
| Tissue infiltrate | 1 | 1 | NA |
| **Overall total** | **1** | **5** | **0** |

All pathologists are board-certified. Pathologist 1 and 2 are from Weill Cornell Medicine and Houston Methodist Hospital, respectively. Animal Treatment 6 was not used to calculate total histological score because the implant site was not easily identifiable.

## References

1. Su, J.T.; Simpson, S.M.; Sung, S.; Tfaily, E.B.; Veazey, R.; Marzinke, M.; Qiu, J.; Watrous, D.; Widanapathirana, L.; Pearson, E.; et al. A Subcutaneous Implant of Tenofovir Alafenamide Fumarate Causes Local Inflammation and Tissue Necrosis in Rabbits and Macaques. *Antimicrob. Agents Chemother.* **2019**, *64*, 775452, doi:10.1128/aac.01893-19.

© 2020 by the authors. Licensee MDPI, Basel, Switzerland. This article is an open access article distributed under the terms and conditions of the Creative Commons Attribution (CC BY) license (http://creativecommons.org/licenses/by/4.0/).
